# Supplementary material for: Dynamic transcriptomic profiles of zebrafish gills in response to zinc supplementation
Source: BMC Genomics. 2010 Oct 11;11:553. doi: 10.1186/1471-2164-11-553 (PMC3091702; doi:10.1186/1471-2164-11-553)
Supplement: Additional file 2 — Interactive Direct Interaction Network representing the molecular interactions between zinc, copper, iron, calcium and proteins encoded by transcripts changed by zinc supplementation. Mini web-site containing index.html and hyperlinked pages in subdirectory describing a Direct Interaction Network automatically generated based on curated interactions contained within the proprietary PathwayArchitect database. Ovals represent proteins and the circles symbolize metal ions. Objects are coloured by their abundance in zebrafish at the time-point they were significantly different from the control is a scale from -4 fold (dark green) to +4 fold (dark red). Where significant differences were found at more than one time-point, the colour overlay shows expression at the first instance. Dark blue squares denote 'binding', and light blue squares 'expression'; green squares stand for 'regulation', green diamonds for 'metabolism', and green circles for 'promoter binding'. Arrow heads indicate directionality of the interaction where annotated. All nodes and edges can be further interrogated by selecting the relative area of the image. [file 1471-2164-11-553-S2.zip › PathwayArchitect Zn xs DIN/517296.html]

# PROTEIN MODIFICATION:

|  |  |
| --- | --- |
| Type | PROTEIN MODIFICATION |
| Effect | None |


---

|  |  |
| --- | --- |
| Score | 0 |


---

|  |  |
| --- | --- |
| Reference Count | 14 |


---

|  |  |
| --- | --- |
| Mechanism | Unknown |


---

|  |  |
| --- | --- |
| Reference:0 || Sentence | "As the protein expression of SERCA IIa and phospholamban seems to be similar in NYHAIV and nonfailing tissue, the reduced Ca(2+)- uptake may result from an altered regulation of these proteins, e.g., reduced phosphorylation of phospholamban or the SERCA IIa." |
| PMID | 8957538 |
| Year | 1996 |
| Species | Human |
| Journal | Basic Res Cardiol |
| RefScore | 1 |
| Source | PArchNLP |
  |
|  |  |


---

|  |  |
| --- | --- |
 Reference:1 || Sentence | "PKC-mediated phosphorylation of phospholamban was fully activated by 1 microM Ca2+ whereas the Ca2+/calmodulin dependent kinase required concentrations in excess of 5 microM Ca2+." |
| PMID | 8700163 |
| Year | 1996 |
| Journal | Mol Cell Biochem |
| RefScore | 1 |
| Source | PArchNLP |
  ||  |  |


---

|  |  |
| --- | --- |
 Reference:2 || Sentence | "However, phosphorylation of phospholamban had no effect on the Ca2+ accumulation of the reconstituted vesicles." |
| PMID | 2935532 |
| Year | 1986 |
| Journal | J Biol Chem |
| RefScore | 0 |
| Source | PArchNLP |
  ||  |  |


---

|  |  |
| --- | --- |
 Reference:3 || Sentence | "Beta-adrenoceptor/cAMP-dependent Ser16-phosphorylation as well as Ca(2+)-dependent Thr17-phosphorylation of phospholamban (PLB) influences SERCA 2a activity and thus myocardial contractility." |
| PMID | 14530977 |
| Year | 2003 |
| Species | Human |
| Journal | Pflugers Arch |
| RefScore | 1 |
| Source | PArchNLP |
  ||  |  |


---

|  |  |
| --- | --- |
 Reference:4 || Sentence | "This endoplasmic reticulum Ca2+ pump in smooth muscle is regulated by cAMP and cGMP via phosphorylation of phospholamban." |
| PMID | 2531511 |
| Year | 1989 |
| Species | Human |
| Journal | Verh K Acad Geneeskd Belg |
| RefScore | 1 |
| Source | PArchNLP |
  ||  |  |


---

|  |  |
| --- | --- |
 Reference:5 || Sentence | "The results suggest a physiological role of the Ca(2+)-calmodulin dependent phospholamban phosphorylation pathway as a mechanism that supports, in part, the beta-adrenergic cardiac relaxant effect." |
| PMID | 8232275 |
| Year | 1993 |
| Species | Rat |
| Journal | Mol Cell Biochem |
| RefScore | 0 |
| Source | PArchNLP |
  ||  |  |


---

|  |  |
| --- | --- |
 Reference:6 || Sentence | "Recent studies have shown that beta 2-adrenergic receptor (beta 2-AR)-stimulated increases in the intracellular Ca2+ (Cai) transient and contraction in cardiac myocytes are dissociated from the increase in adenosine 3',5'-cyclic monophosphate (cAMP) level and are not accompanied by an increase in phospholamban phosphorylation, an acceleration in relaxation, or a reduction in myofilament Ca2+ response." |
| PMID | 9321856 |
| Year | 1997 |
| Species | Rat |
| Journal | Am J Physiol |
| RefScore | 1 |
| Source | PArchNLP |
  ||  |  |


---

|  |  |
| --- | --- |
 Reference:7 || Sentence | "In summary, our results reflect a cross-talk between beta -adrenoceptor stimulation and intracellular Ca(2+)at the level of CaMKII-mediated phospholamban phosphorylation in neonatal rat cardiomyocytes." |
| PMID | 11112993 |
| Year | 2000 |
| Species | Rat |
| Journal | J Mol Cell Cardiol |
| RefScore | 1 |
| Source | PArchNLP |
  ||  |  |


---

|  |  |
| --- | --- |
 Reference:8 || Sentence | "We postulate that Ca(2+)transport systems of the sarcoplasmic reticulum are critical determinants for the activation of CaMKII that catalyzes phosphorylation of phospholamban." |
| PMID | 11112993 |
| Year | 2000 |
| Species | Rat |
| Journal | J Mol Cell Cardiol |
| RefScore | 1 |
| Source | PArchNLP |
  ||  |  |


---

|  |  |
| --- | --- |
 Reference:9 || Sentence | "Acceleration of cardiac relaxation upon beta adrenergic stimulation is due, in part, to enhancement in the rate of Ca2+ sequestration by the sarcoplasmic reticulum (SR) Ca2+ pump resulting from cAMP-mediated phosphorylation of the SR protein phospholamban." |
| PMID | 2366595 |
| Year | 1990 |
| Species | Rat |
| Journal | Mech Ageing Dev |
| RefScore | 1 |
| Source | PArchNLP |
  ||  |  |


---

|  |  |
| --- | --- |
 Reference:10 || Sentence | "Enhanced phosphorylation of PLB by membrane-bound Ca(2+)-CaM protein kinase also appeared to be most pronounced in biopsy from a patient with a very high CTG expansion, as was the overall 'slow-to-fast' transformation of the same muscle biopsy." |
| PMID | 8845717 |
| Year | 1996 |
| Species | Human |
| Journal | Neuromuscul Disord |
| RefScore | 1 |
| Source | PArchNLP |
  ||  |  |


---

|  |  |
| --- | --- |
 Reference:11 || Sentence | "The stimulation of Ca2+ uptake associated with cAMP-dependent phosphorylation of phospholamban could be reproduced by incubation of microsomes with a monoclonal antibody to phospholamban." |
| PMID | 1323264 |
| Year | 1992 |
| Species | Human |
| Journal | Basic Res Cardiol |
| RefScore | 1 |
| Source | PArchNLP |
  ||  |  |


---

|  |  |
| --- | --- |
 Reference:12 || Sentence | "Thus regulatory steps include PLN dissociation, PLN/SERCA2a inhibitory association, and PLN/SERCA2a dissociation induced by phosphorylation of PLN (in the noninhibitory cytoplasmic domain) or by binding of Ca2+ by SERCA2a (in the inhibitory transmembrane domain)." |
| PMID | 10603934 |
| Year | 1998 |
| Journal | Ann N Y Acad Sci |
| RefScore | 1 |
| Source | PArchNLP |
  ||  |  |


---

|  |  |
| --- | --- |
 Reference:13 || Sentence | "In the adult myocardium the Ca2+ uptake and release functions of the sarcoplasmic reticulum (SR) are known to be regulated by a membrane-associated Ca2+-calmodulin-dependent protein kinase (CaM kinase) which phosphorylates the Ca2+-pumping ATPase (Ca2+ pump), Ca2+ release channel (ryanodine receptor) and the Ca2+ pump-regulatory protein, phospholamban." |
| PMID | 9040054 |
| Year | 1997 |
| Journal | J Mol Cell Cardiol |
| RefScore | 1 |
| Source | PArchNLP |
  |


---

|  |  |
| --- | --- |
